# Supplementary material for: Synergistic enhancement of AAV gene delivery in 2D cells and 3D organoids using polybrene and hydroxychloroquine
Source: PLoS One. 2025 Nov 14;20(11):e0336164. doi: 10.1371/journal.pone.0336164 (PMC12617951; doi:10.1371/journal.pone.0336164)
Supplement: S1 File — Step-by-step protocol, also available on protocols.io (PDF) [file pone.0336164.s001.pdf]

Sep 30, 2025 Version 2

Synergistic Enhancement of AAV Gene Delivery in 2D Cells and 3D Organoids Using Polybrene and Hydroxychloroquine V.2

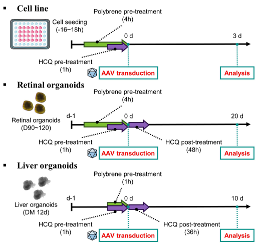

DOI
dx.doi.org/10.17504/protocols.io.4r3l29mjjv1y/v2

Hyeon-Jin Na[1,2], Yongbo Shin[2,3], Seung-Hyun Kim[1], Seung pil Jang[1], Myung Jin Son[2,3], Yong Min Choi[1,2], Hyeon Gyeol Jeon[2,3], Ok-Seon Kwon[1,2], Kyung-Sook Chung[1,2]

[1]Center for Gene and Cell Therapy, Korea Research Institute of Bioscience and Biotechnology (KRIBB), Republic of Korea;
[2]Department of Advanced Bioconvergence, Korea University of Science & Technology (UST), Republic of Korea;
[3]Stem Cell Convergence Research Center, Korea Research Institute of Bioscience and Biotechnology, Daejeon, Republic of Korea

Na HyeonJin

Hyeon-Jin Na
Korea Research Institute of Bioscience and Biotechnology

OPEN ACCESS

DOI: https://dx.doi.org/10.17504/protocols.io.4r3l29mjjv1y/v2

Protocol Citation: Hyeon-Jin Na, Yongbo Shin, Seung-Hyun Kim, Seung pil Jang, Myung Jin Son, Yong Min Choi, Hyeon Gyeol Jeon, Ok-Seon Kwon, Kyung-Sook Chung 2025. Synergistic Enhancement of AAV Gene Delivery in 2D Cells and 3D Organoids Using Polybrene and Hydroxychloroquine. **protocols.io** https://dx.doi.org/10.17504/protocols.io.4r3l29mjjv1y/v2Version created by **Hyeon-Jin Na**

License: This is an open access protocol distributed under the terms of the **Creative Commons Attribution License**, which permits unrestricted use, distribution, and reproduction in any medium, provided the original author and source are credited

Protocol status: Working
We use this protocol and it's working

Created: October 28, 2024

Last Modified: September 30, 2025

Protocol Integer ID: 228092

Keywords: Polybrene, Hydroxychloroquine sulfate (HCQs), Organoids, Virus transduction, Adeno-associated virus(AAV), Gene Therapy, synergistic enhancement of aav gene delivery, aav gene delivery, significant challenges for efficient gene delivery, efficient gene delivery, gene delivery, advancing gene therapy application, treatment with virus, gene therapy application, aav transduction, 3d organoid, liver organoid model, using polybrene, liver organoid system, enabled organoid, aav vector, hydroxychloroquine, current limitations in organoid, evaluating aav vector, 2d cell, viral entry, endosomal processing, polybrene

Funders Acknowledgements:
National Research Council of Science & Technology
Grant ID: GTL24022-000
Korea Research Institute of Bioscience and Biotechnology (KRIBB) Research Initiative Program
Grant ID: KGM5362521
National Research Foundation of Korea (NRF)
Grant ID: RS-2024-00352135

Abstract

Recent advances in three-dimensional (3D) culture platforms have enabled organoids to serve as physiologically relevant models for recapitulating human biology and assessing therapeutic efficacy and toxicity. Despite their promise, their complex architecture presents significant challenges for efficient gene delivery, thereby limiting their broader application in drug discovery and translational research. To overcome this challenge, we developed a sequential treatment strategy that combines polybrene (PB), which facilitates viral entry, and hydroxychloroquine (HCQ), which modulates endosomal processing. By applying PB as a pre-treatment and HCQ as a post-treatment, we achieved an approximate 1.3- to 2-fold increase in adeno-associated virus (AAV) transduction efficiency in both retinal and liver organoid models compared to single-agent treatments, and a 1.7- to 2.5-fold increases compared to treatment with virus alone. Importantly, this combinatorial treatment preserved cellular integrity, as confirmed by minimal TUNEL assay and high overall viability. Our findings demonstrate that sequential administration PB and HCQ significantly improves AAV transduction in 3D retinal and liver organoid systems, offering a robust method to improve gene delivery. This approach not only overcomes current limitations in organoid-based research but also supports the development of more predictive platforms for evaluating AAV vectors and advancing gene therapy application.

Guidelines

- The stock concentration may vary depending on experimental requirements.
- Sterilize 1.5 mL micro-centrifuge tubes prior to use.
- The cell culture process can be optimized based on cell line characteristics, such as trypsin-EDTA incubation time.
- The optimal maturation day for retinal organoids is between day 90 to day 120.

> **Note**
>
> Differentiation day may vary according to the experimental objectives.

- For chemical treatment, add the diluted chemical directly to the media, adjusting for the buffer volume.
- When conducting multiple reactions, it is advisable to prepare a master mix that includes a 10% excess to account for pipetting errors.

Materials

- Laboratory Equipment

1. CO2 incubator, clean bench, Vacuum pump
2. Pipette, pipette aid, table-top centrifuge, centrifuge, vortex
3. Refrigerator (4 °C), freezer(-20 °C) and deep-freezer (-80 °C)
4. Bright-field microscope, ZEISS confocal microscope (LSM800), Cytation 5
5. Real-time PCR, Cryostat microtome
6. GraphPad Prism, ZEISS blue software, BioTek Gen5
7. Personal Protective Equipment (PPE) at a minimum laboratory coat with fitted sleeves, latex or nitrile gloves and safety glasses

- Materials

A
50-mL, 25-mL, 10-mL, 5-mL, 2-mL sterile pipette
1000-µl, 200-µl, 10-µl pipette tips
50-mL, 15-mL conical tubes
1.5-mL sterile micro-centrifuge tubes
35-mm, 100-mm cell culture dishes
24-well, 48-well cell culture plates
96-well v-bottom ultra-low attachment plates

- Reagents for stock solution preparations

Reagent	Company	Cat #
DPBS, no calcium no magnesium	ThermoFisher	14190144
UltraPure DNase/RNase-Free Distilled Water	Invitrogen	10977015
Dimethyl Sulfoxide (DMSO), cell culture reagent	Santa Cruz	sc-358801

DPBS, no calcium, no magnesium **Thermo Fisher Catalog #**14190144

UltraPure™ DNase/RNase-Free Distilled Water **Thermo Fisher Scientific Catalog #**10977015

Dimethyl Sulfoxide (DMSO), cell culture reagent (CAS 67-68-5) **Santa Cruz Catalog #**sc-358801

- Reagent for cell line culture

Reagent	Company	Cat #
DMEM	ThermoFisher	11995-065
Fetal Bovine Serum	ThermoFisher	14000044
Penicillin/Streptomycin	ThermoFisher	15140-122
Trypsin-EDTA(0.5%), no phenol red	ThermoFisher	15400054
PBS pH 7.4(10X)	ThermoFisher	70011-044

DMEM **Gibco - Thermo Fisher Scientific Catalog** #11995065

Penicillin-Streptomycin (10,000 U/mL) **Thermo Fisher Scientific Catalog** #15140122

Trypsin-EDTA (0.5%) no phenol red **Thermo Fisher Scientific Catalog** #15400054

10X PBS pH 7.4 **Thermo Fisher Scientific Catalog** #70011044

- Reagent for hPSCs and Organoid culture

Reagent	Company	Cat #
mTeSR1 complete kit	STEMCELL	ST85850
DMEM/F12	ThermoFisher	11320-033
IMDM	ThermoFisher	12440053
Ham F12	ThermoFisher	11765054
dPBS	ThermoFisher	14190144
Matrigel	Corning	354277
Knockout Serum replacement	ThermoFisher	10828028
Fetal Bovine Serum	ThermoFisher	14000044
ReLeSR	STEMCELL	ST05872
Gentle cell dissociation reagent	STEMCELL	ST07174
N2 supplement	ThermoFisher	17504044
GlutaMAX	ThermoFisher	35030061
Chemically Defined Lipid Concentrate	ThermoFisher	11905031
Penicillin/Streptomycin	ThermoFisher	15140-122
Cell Recovery solution	Corning	354253
Trypsin-EDTA	Thermo	25200056
Y-27632	Tocris	4423
Advanced DMEM/F12	Thermo Fisher	12634028
HEPES	Thermo Fisher	15630080
B27 Supplement with Vitamin A	Thermo Fisher	17504044
N-Acetylcysteine	Sigma-Aldrich	A9165
A83-01	Tocris	2939
DAPT	Sigma-Aldrich	D5942
Dexamethasone	Sigma-Aldrich	D4920
[Leu]-Gastrin I human	Sigma-Aldrich	G9145
Human HGF	Peprotech	100-39
Human EGF	Peprotech	AF-100-15
Human BMP7	Peprotech	120-03
Human FGF19	Peprotech	100-32

mTeSR™1 **STEMCELL Technologies Inc. Catalog #**85850

DMEM/F-12 **Thermo Fisher Scientific Catalog #**11320033

IMDM **Thermo Fisher Catalog #**12440053

Hams F12 **Thermo Scientific Catalog #**11765054

DPBS **Thermo Fisher Scientific Catalog #**14190144

Corning® Matrigel® **Corning Catalog #**354277

KnockOut™ Serum Replacement **Thermo Fisher Catalog #**10828028

ReLeSR **STEMCELL Technologies Inc. Catalog #**05872

Gentle Cell Dissociation Reagent **STEMCELL Technologies Inc. Catalog #**07174

Chemically Defined Lipid Concentrate **Thermo Fisher Catalog #**11905031

Penicillin/Streptomycin **Invitrogen - Thermo Fisher Catalog #**15140-122

Cell Recovery solution **Corning Catalog #**354253

Trypsin-EDTA (0.25%), phenol red **Thermo Fisher Catalog #**25200056

CHIR 99021 **Tocris Catalog #**4423

Advanced DMEM/F-12 **Thermo Fisher Catalog #**12634028

HEPES 1M **Thermo Fisher Scientific Catalog #**15630080

N-Acetyl-L-cysteine **Merck MilliporeSigma (Sigma-Aldrich) Catalog #**A9165

A 83-01 **Tocris Catalog #**2939

DAPT **Merck MilliporeSigma (Sigma-Aldrich) Catalog #**D5942

[Leu15]-Gastrin I human **Merck MilliporeSigma (Sigma-Aldrich) Catalog #**G9145

Gibco™ Human HGF Recombinant Protein, PeproTech® **Fisher Scientific Catalog #**100-39-10UG

Epidermal growth factor **peprotech Catalog #**AF-100-15

Human BMP-7 Recombinant Protein, PeproTech® **Thermo Fisher Scientific Catalog #**120-03P-10UG

Human FGF-19, Animal-Free Recombinant Protein, PeproTech® **Thermo Fisher Scientific Catalog #**AF-100-32-25UG

- Reagent for AAV transduction

Reagent	Company	Cat #
Hydroxychloroquine (HCQ)	Tocris	5648
Polybrene (Hexadimethrine bromide)	Sigma	107689

Hydroxychloroquine sulfate **Tocris Catalog #**5648

Hexadimethrine bromide **Merck MilliporeSigma (Sigma-Aldrich) Catalog #**107689

- Preparation of Medium

Medium	Composition
10DMEM medium	10% Fetal Bovine Serum, 1% Penicillin and Streptomycin supplied in DMEM media
mTeSR1 medium	100ml of mTeSR1 5X supplement supplied in mTeSR1 basal media
gfCDM Neural induction medium	10% Knockout Serum replacement, 1% Chemically Defined Lipid Concentrate, 450 µM monothioglycerol, 1% Penicillin and streptomycin supplied in basal media (IMDM:Ham F12 (1:1))
Neural retina maturation medium	10% Fetal Bovine Serum, 1% GlutaMAX, 1% N2, 0.5µM Retinoic acid, 100 µM Taurine supplied in DMEM/F12 media
Virus medium	10% Fetal Bovine Serum, 1% GlutaMAX, 1% N2, 1% Penicillin and Streptomycin supplied in DMEM/F12 media
Liver organoid differentiation medium(DM)	1% Penicillin/Streptomycin (P/S), 1% GlutaMAX, 1% HEPES, 1X N2 Supplement, 1X B27 Supplement with Vitamin A, N-Acetylcysteine 1 mM, A83-01 0.5 µM, DAPT 10 µM, Dexamethasone 3 µM, [Leu]-Gastrin I human 10 µM, Human HGF 25 ng/ml, Human EGF 50 ng/ml, Human BMP7 25 ng/ml, Human FGF19 100 ng/ml supplied in Advanced DMEM/F12 media
Basal medium	1% Penicillin/Streptomycin (P/S) supplied in Advanced DMEM/F12 media

- Cell lines

Cell line	Description	Company, Cat #
HEK293FT	fast-growing, highly transfectable clonal isolate derived from human embryonal kidney cells	ThermoFisher, R70007
661W	immortalized cone photoreceptor cell line derived from the retinal tumor of a mouse	kindly provided by Prof. Muayyad Al-Ubaidi, University of Houston
H9	Human embryonic stem cell (hESC)	WI Cell, WA09

- Material for analysis preparation

Reagent	Company	Cat #
4% Paraformaldehyde	Biosesang	PC2031-050-00
Tritone X-100	Sigma-Aldrich	X100
Tween-20	Sigma-Aldrich	P7949
Sucrose	LPS Solution	SUC05
Tissue-Tek O.C.T. Compound	SAKURA	4583
Aqueous Mounting Medium	Vector Laboratories, Inc	H-5501
ImmEdge Pen	Vector Laboratories, Inc	H-4000

Reagent	Company	Cat #
In Situ Cell Death Detection Kit, Fluorescein	Roche	11684795910
Cell Counting Kit-8	DOJINDO Laboratories	CK04

Safety warnings

Proper Personal Protective Equipment (PPE) needed; a minimum laboratory coat with fitted sleeves, latex or nitrile gloves and safety glasses

Formulation of chemical

1 **Polybrene**

1.1 Prepare autoclaved tertiary distilled water.

1.2 Dissolve the polybrene powder in tertiary distilled water to make a final concentration of 5 mg/ml ~ 10 mg/ml .

1.3 Filter polybrene solution with 0.22 µm filter.

1.4 Aliquot polybrene stock into 1.5 mL sterile micro-centrifuge tubes.

2 **Hydroxychloroquine**

2.1 Prepare autoclaved tertiary distilled water.

2.2 Dissolve the HCQ tablet in tertiary distilled water to make a final concentration of [M] 10 millimolar (mM) .

2.3 Filter HCQ solution with 0.22 µm filter.

2.4 Aliquot HCQ stock into 1.5 mL sterile micro-centrifuge tubes.

Preparation of cell line

3 **Sub-culture of normal cell line**

3.1 Aspirate culture media and wash with 1X PBS.

3.2 Add 1 mL of 0.05% Trypsin-EDTA in the culture plate and incubate culture plate in 5% CO_2, 37 °C culture incubator for approximately 00:02:00 . 2m

3.3 Add 1 mL of 10DMEM medium and detach the cancer cell by tapping the plate with hands.

3.4 Transfer the detached cells to 15 mL conical tube and centrifuge it under 1000 rpm, 00:03:00 . 3m

3.5 Aspirate supernatant and add 1 mL of 10DMEM medium to resuspend cells.

3.6 Seed a ratio of 1/5 of the cells into a new 100-mm cell culture dish with 10DMEM medium.

4 Seeding of cell lines

4.1 Repeat steps in **Sub-culture of cell lines** [go to step #3.1 go to step #3.5].

4.2 Dilute 100 µL to 1/10 by adding 900 µL of fresh media, making a final volume of 1 mL .

4.3 Mix 20 µL of 1/10 diluted cell resuspension with 20 µL of Trypan Blue in a 1.5 mL sterile micro-centrifuge tube.

4.4 Clean a hematocytometer and load 10 µL of the sample in each side.

4.5 Count the cells and seed appropriate number of cells into a new 48-well plate.

5 Sub-culture of human Embryonic Stem Cells

5.1 Human embryonic stem cells (hESCs; H9) were maintained under feeder-free conditions using mTeSR1 medium on Matrigel-coated culture plates at 37 °C with 5% CO_2

5.2 The culture medium was refreshed on a daily basis.

5.3 For routine passaging, colonies were dissociated into small clumps with ReLeSR and reseeded at a split ratio of 1:10–1:20 approximately once per week.

Preparation of organoids

6 **Retinal organoids**

6.1 Perform retinal organoids differentiation according to previously validated protocols.

Citation

Na HJ, Kwon JE, Kim SH, Ahn J, Kwon OS, Chung KS (2024)
. Human Pluripotent Stem Cell-Derived Retinal Organoids: A Viable Platform for Investigating the Efficacy of Adeno-Associated Virus Gene Therapy..
International journal of stem cells.

https://doi.org/10.15283/ijsc23071 LINK

6.2 Prepare retinal organoids and transfer retinal organoids to a new 96 well U-bottom or flat-bottom low attachment plate.

6.3 Change medium into fresh pre-warmed virus medium up to 200 µL .

7 **Liver organoids**

7.1 Perform liver organoids differentiation according to previously validated protocols.

Citation

Mun SJ, Hong YH, Shin Y, Lee J, Cho HS, Kim DS, Chung KS, Son MJ (2023)
. Efficient and reproducible generation of human induced pluripotent stem cell-derived expandable liver organoids for disease modeling..
Scientific reports.

https://doi.org/10.1038/s41598-023-50250-w LINK

Citation

Mun SJ, Ryu JS, Lee MO, Son YS, Oh SJ, Cho HS, Son MY, Kim DS, Kim SJ, Yoo HJ, Lee HJ, Kim J, Jung CR, Chung KS, Son MJ
(2019)
. Generation of expandable human pluripotent stem cell-derived hepatocyte-like liver organoids..
Journal of hepatology.

https://doi.org/10.1016/j.jhep.2019.06.030 LINK

7.2 Prepare liver organoid differentiation medium (DM) and basal medium, and store them at 4 °C until use.

7.3 Dilute Matrigel in cold DPBS at a 1:100 ratio and coat a flat-bottom 24-well plate for 01:00:00 in a 37 °C incubator. (Keep the Matrigel On ice at 4 °C .) 1h

7.4 Harvest the organoid embedded in Matrigel dome using a 1000 µL pipette tip, transfer it into microcentrifuge tubes, and briefly spin down using a mini centrifuge to pellet the organoids.

7.5 Remove the supernatant, resuspend the organoid pellet in basal medium, and briefly spin down again using a mini centrifuge.

7.6 After removing the supernatant, add cold Cell Recovery Solution (60 µL per dome) and incubate the cultures in Cell Recovery solution at 4 °C for approximately 10m

00:10:00 .

7.7 Briefly centrifuge the cultures to separate the organoids from the Cell Recovery solution. Remove the supernatant and wash the cultures with cold DPBS twice.

7.8 Remove the supernatant and resuspend the organoids in DM (100 µL per well of a flat-bottom 24-well plate).

7.9 Add 400 µL of fresh DM to each well of the Matrigel-coated 24-well plate (go to step #7.3), and seed the 12-15 organoids per well by adding 100 µL of organoid suspension (as prepared in go to step #7.8). (Additionally, prepare three wells of organoids for cell counting, to be used in go to step #11.1 below.)

7.10 To anchor the organoids (but not fully attached) to the bottom of the Matrigel-coated plate, incubate the organoids for 04:00:00 - 06:00:00 in a 37 °C , 5% CO_2 incubator. 10h

Treatment of chemicals

8 **Treatment of polybrene**

8.1 16:00:00 to 18:00:00 after seeding an appropriate number of cells per well, treat with the polybrene at a concentration of 5 µg/ml ~ 10 µg/ml .

8.2 In the case of organoids, on the day organoids are transferred, polybrene can be treated at a concentration of of 5 µg/ml ~ 15 µg/ml .

8.3 About 01:00:00 to 04:00:00 after, AAV can be transduced in either cells or organoids.

8.4

> **Note**
>
> Cell viability under these treatment conditions was assessed and found to remain above 80% (see *Analysis for cell viability* section).

9 **Treatment of HCQ**

9.1 16:00:00 to 18:00:00 after seeding an appropriate number of cells per well, treat with the HCQ at a concentration of 15 µM .

9.2 In the case of organoids, on the day organoids are transferred, HCQ at a concentration of 10 µM , 15 µM , 20 µM .

9.3 01:00:00 after, AAV can be transduced in either cells or organoids.

9.4 In the sequential treatment protocol for organoids, HCQ is applied for 36:00:00 to 48:00:00 post-viral transduction.

9.5

> **Note**
>
> Cell viability under these treatment conditions was assessed and found to remain above 80% (see *Analysis for cell viability* section).

Virus transduction

10 **Transduction of AAV to cell line and retinal organoids**

10.1 Observe the morphology and condition of cells.

10.2 Dilute AAV in 1X PBS at the desired MOI. (Ex. 2×10^4 *vg*/cell or 1×10^{10} *vg*/organoid)

> Note
>
> AAV stock needs to be diluted with the appropriate dilution factor. The Volume of transduced AAV should be over 1 µL to avoid pipetting errors.

10.3 Transduce the diluted AAV into the cells.

10.4 Incubate in a 37 °C , 5% CO_2 incubator.

10.5 In the case of retinal organoids, Incubate for 15 days in a 37 °C , 5% CO_2 incubator. Change the medium every 2 days.

11 Transduction of AAV to liver organoids

11.1 To calculate the treatment concentration of AAVs, transfer organoids from three wells (n=3) as prepared in go to step #7.9 into microcentrifuge tubes, and briefly spin down using a mini centrifuge.

11.2 Remove the supernatant, resuspend the organoid pellet in basal medium, and spread the organoid suspension onto the cell culture dish.

11.3 To dissociate the organoids into single cells for counting, mechanically chop the organoids using a surgical blade.

11.4 Transfer the cells into microcentrifuge tube and incubate them with Trypsin-EDTA for 00:10:00 in a 37 °C incubator. 10m

> Note
>
> Every 00:05:00 , pipette up and down using a 200 µL pipette tip to fully dissociate the cells into a single-cell suspension.

11.5 Count the number of cells using a standard hematocytometer or automated cell counter. (10–12 organoids per well is approximately equivalent to $1×10^5$ cells per well.)

11.6 Change the medium to 250 µL of DM supplemented with 10 µM Y-27632 and the appropriate concentration of AAVs. (Ex, Use $1×10^1$ to $1×10^3$ *vg*/cell for evaluating AAV cell entry, and $1×10^6$ *vg*/cell for transgene expression analysis.)

11.7 Incubate the organoids on a bi-directional rocker in a 37 °C , 5% CO_2 incubator. 1d

11.8 For AAV entry analysis, transfer the AAV-transduced organoids (go to step #11.7) from the matrigel-coated plate into microcentrifuge tubes and wash organoids with DPBS three times.

11.9 The organoid pellet can be used for next generation sequencing (NGS) analysis or stored at -80 °C for later analysis.

11.10 For further incubation of the AAV-transduced organoids (go to step #11.8), Resuspend organoids with Matrigel (30 µL per dome) and add the Matrigel droplet to the center of each well in a new flat-bottom 24-well plate.

11.11 Incubate the cells for 5–10 minutes in a 37 °C incubator until the Matrigel is solidified.

11.12 Overlay the droplet with DM (500 µL per well for a 24-well plate) and incubate for 3–10 days in
a 37 °C , 5% CO2 incubator. Change the medium every 2 days.

Analysis for cell viability

1h

12 **Sample preparation for cell viability assay**

12.1 Count and seed the cells to multi-well plates.

> Note
>
> Make sure to place a well of only media to measure the background.

12.2 Add the reagent at a final volume corresponding to 1/10 of the culture medium. (In our case, this was 20 µL per well in a 48-well plate containing 200 µL of medium.)

12.3 Incubate culture plate in 37 °C , 5% CO_2 incubator for approximately 01:00:00 . 1h

12.4 Transfer 150 µL of medium containing the reagent into each well of a 96-well plate.

12.5 Measure the absorbance at 450 nm with a microplate reader.

12.6 Enter the absorbance reading from each well in the equation below to calculate the cell survival rate.

$$Survival\ rate\ (\%) = (A_{sample} - A_{blank})/(A_{negative\ control} - A_{blank}) \times 100$$

Analysis for protein expression with Flow cytometry

13 **Sample preparation for Flow cytometry**

13.1 Observe the morphology and condition of 2D cells.

13.2 Aspirate culture media and wash with 1X PBS.

13.3 Add 100 µL of 0.05% Trypsin-EDTA in the culture plate and incubate culture plate in 37 °C , 5% CO_2 incubator for approximately 00:02:00 . 2m

13.4 Add 100 µL of 2% FBS in 1X PBS and detach the cells by tapping the plate with hands.

13.5 Pipette and transfer the detached cells to a 1.5 mL sterile micro-centrifuge tube.

13.6 Add 300 µL of 2% FBS in 1X PBS and strain using a 5 mL polystyrene round-bottom tube with a cell strainer cap.

14 **Flow cytometry**

14.1 Run the flow cytometry software.

14.2 Load the samples one by one.

14.3 Analyze the data.

Analysis for protein expression with immunofluorescence chemistry

4h

15 **Sample preparation for immunofluorescence staining**

15.1 Collect retinal organoid samples using a 2 mL pipette aid and transfer them to 1.5 mL sterile micro-centrifuge tube.

> Note
>
> In the case of liver organoids, follow the steps (go to step #7.4 go to step #7.7)

15.2 Wash the samples twice with 1X PBS.

15.3 Remove the 1X PBS and add 1 mL of 4% paraformaldehyde (PFA), incubating for 01:00:00 at Room temperature .

1h

15.4 Remove the 4% PFA and wash the samples 3 times with 1X PBS.

15.5 Incubate the samples in 15% sucrose in 1X PBS for 01:00:00 at Room temperature .

1h

15.6 Remove the 15% sucrose and add 30% sucrose in 1X PBS, incubating Overnight at 4 °C . 1h

15.7 Embed the samples in OCT compound and freeze them in a deep-freezer Overnight at -80 °C . 1h

15.8 Section the samples at a thickness of 10 µm using a cryotome and mount three sections per silane-coated slide.

Note

The slides should be stored -20 °C unless staining is performed immediately.

16 **Immunofluorescence staining of slides**

16.1 Wash the slide with tertiary distilled water to remove remaining OCT compound around the samples.

16.2 Gently remove excess moisture around the samples using Kimtech wipes.

Note

The samples must not be dried.

16.3 Create a barrier around the samples using a barrier pen.

16.4 Add blocking and permeabilization buffer consisting of 5% FBS with 0.1% Triton X-100 in 1X PBS and incubate for at least 00:30:00 at Room temperature in a humid chamber. 30m

16.5 Dilute the primary antibody with the appropriate dilution factor in 1% FBS in 1X PBS.

16.6 Aspirate the blocking and permeabilization buffer and apply the diluted primary antibody solution, incubating the slides in the humid chamber Overnight at 4 °C . 30m

16.7 Wash the slides in 0.1% PBST containing 1% FBS five times, for 00:10:00 each. 10m

16.8 Dilute the secondary antibody and Hoechst with the appropriate dilution factor in the same solution used for the primary antibody.

16.9 Gently remove excess moisture around samples and apply the diluted secondary antibody, incubating the slides in the humid chamber for 02:00:00 at Room temperature . 2h

16.10 Wash the slides in 0.1% PBST containing 1% FBS five times, for 00:10:00 each. 10m

16.11 Gently remove excess moisture around the samples.

16.12 Apply 1~2 drops of mounting medium on each sample and cover with a cover glass.

16.13 Let the slides dry in a dark place Overnight at Room temperature . 10m

17 **TUNEL assay of slides**

17.1 Follow the manual of In Situ Cell Death Detection Kit, Fluorescein from Roche.

18 **Imaging slides**

18.1 Run the imaging software.

18.2 Load the slides upside-down, ensuring cover glass faces microscope lenses.

18.3 Capture images at 10X to 40X magnification.

Protocol references

1. Mun SJ, Ryu JS, Lee MO, Son YS, Oh SJ, Cho HS, et al. Generation of expandable human pluripotent stem cell-derived hepatocyte-like liver organoids. J Hepatol. 2019;71(5):970-85.
2. Mun SJ, Hong Y-H, Shin Y, Lee J, Cho H-S, Kim D-S, et al. Efficient and reproducible generation of human induced pluripotent stem cell-derived expandable liver organoids for disease modeling. Scientific Reports. 2023;13(1):22935.
3. Moon HR, Mun SJ, Kim TH, Kim H, Kang D, Kim S, et al. Guidelines for Manufacturing and Application of Organoids: Liver. Int J Stem Cells. 2024;17(2):120-9.
4. Na HJ, Kwon JE, Kim SH, Ahn J, Kwon OS, Chung KS. Human Pluripotent Stem Cell-Derived Retinal Organoids: A Viable Platform for Investigating the Efficacy of Adeno-Associated Virus Gene Therapy. Int J Stem Cells. 2024;17(2):204-11.

Citations

Step 6.1

Na HJ, Kwon JE, Kim SH, Ahn J, Kwon OS, Chung KS. Human Pluripotent Stem Cell-Derived Retinal Organoids: A Viable Platform for Investigating the Efficacy of Adeno-Associated Virus Gene Therapy.
https://doi.org/10.15283/ijsc23071

Step 7.1

Mun SJ, Ryu JS, Lee MO, Son YS, Oh SJ, Cho HS, Son MY, Kim DS, Kim SJ, Yoo HJ, Lee HJ, Kim J, Jung CR, Chung KS, Son MJ. Generation of expandable human pluripotent stem cell-derived hepatocyte-like liver organoids.
https://doi.org/10.1016/j.jhep.2019.06.030

Step 7.1

Mun SJ, Hong YH, Shin Y, Lee J, Cho HS, Kim DS, Chung KS, Son MJ. Efficient and reproducible generation of human induced pluripotent stem cell-derived expandable liver organoids for disease modeling.
https://doi.org/10.1038/s41598-023-50250-w
