# Supplementary material for: Synergistic enhancement of AAV gene delivery in 2D cells and 3D organoids using polybrene and hydroxychloroquine
Source: PLoS One. 2025 Nov 14;20(11):e0336164. doi: 10.1371/journal.pone.0336164 (PMC12617951; doi:10.1371/journal.pone.0336164)
Supplement: S1 Table — Cell viability of HEK293T cells treated with PB (10 μg/mL, A) or HCQ (15 μM, B) was measured at the indicated time points using the CCK-8 assay. Data are presented as mean ± SD relative to untreated controls (0 hr, set as 100%; n = 4). Statistical significance was determined using one-way ANOVA with Tukey’s multiple comparisons test; all treatment groups showed p < 0.0001 compared to control, as denoted by the “&” symbol in the table. (DOCX) [file pone.0336164.s003.docx]

| Treatment | PB(10μg/mL) | SD | Significance vs. control |
| --- | --- | --- | --- |
| 0(control) | 100 | ±1.784578 | - |
| 12hr | 84.1619^&^ | ±0.438214 | P<0.0001 |
| 24hr | 83.5201^&^ | ±0.862242 |  |
| 48hr | 82.5421^&^ | ±1.612592 |  |

A

| Treatment | HCQ(15μM) | SD | Significance vs. control |
| --- | --- | --- | --- |
| 0(control) | 100 | ±1.784578 | - |
| 12hr | 90.9291^&^ | ±1.707361 | P<0.0001 |
| 24hr | 92.3116^&^ | ±1.051635 |  |
| 48hr | 83.9421^&^ | ±0.251954 |  |

B

^&^ indicates P < 0.0001 compared to NC
