# Supplementary figures and images for: Synergistic enhancement of AAV gene delivery in 2D cells and 3D organoids using polybrene and hydroxychloroquine
Source: PLoS One. 2025 Nov 14;20(11):e0336164. doi: 10.1371/journal.pone.0336164 (PMC12617951; doi:10.1371/journal.pone.0336164)

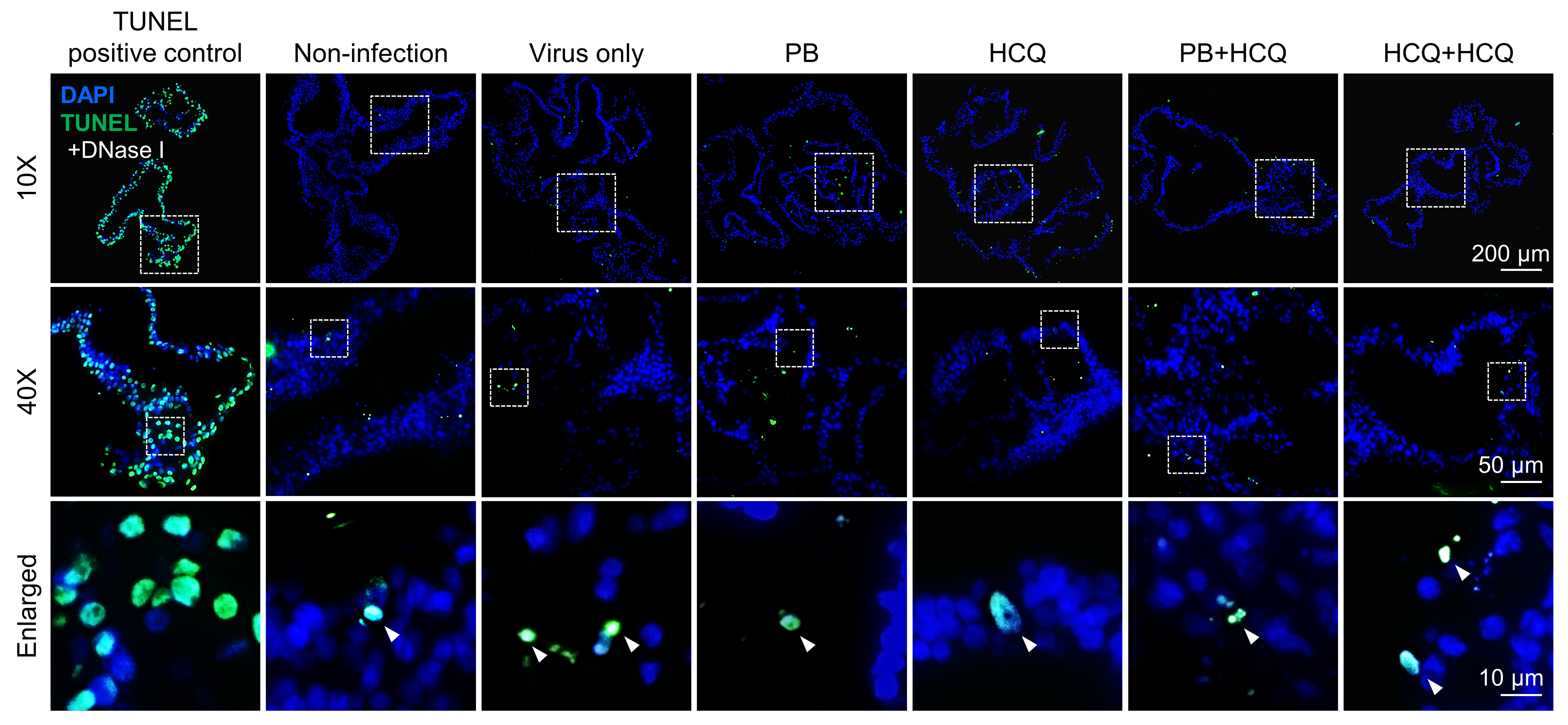

Supplement: S1 Fig — TUNEL staining was performed on liver organoids at day 10 post-transduction. As a positive control, organoids were treated with DNase I prior to staining. TUNEL-positive cells are shown in green, and nuclei are counterstained with DAPI (blue). Magnified views of the areas outlined by white dashed boxes are shown at the bottom of each panel. White arrowheads indicate TUNEL-positive nuclei. Scale bars: 200 μm (top), 50 μm (middle), 10 μm (bottom). (TIF) [file pone.0336164.s004.tif]

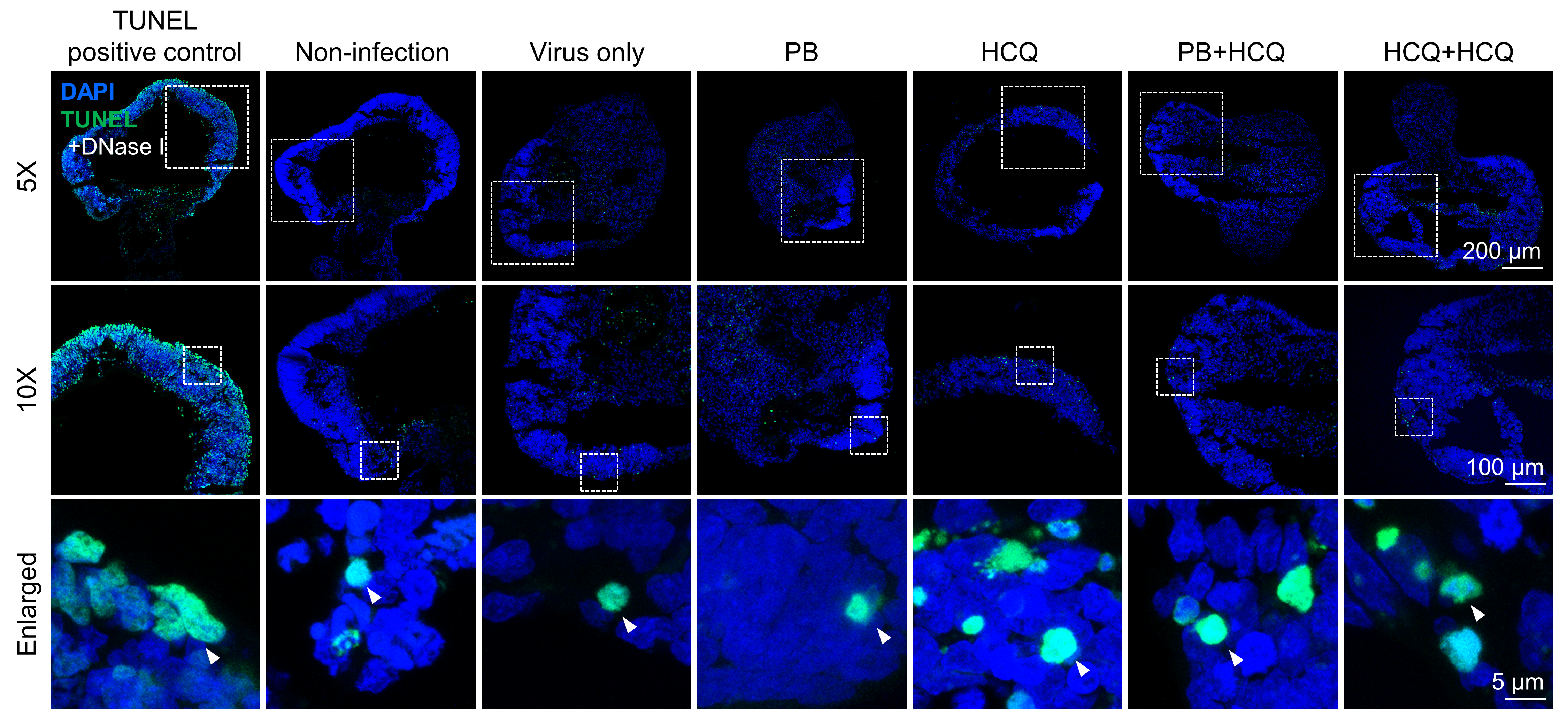

Supplement: S2 Fig — Positive control images of DNase I-treated retinal organoids confirm the validity of the TUNEL assay. Enlarged (40×) images highlight the co-localization of DAPI-stained cell nuclei with TUNEL-positive signals. Magnified views of the regions indicated by white dashed boxes are displayed at the bottom of each panel. Scale bars: 200 μm (top), 100 μm (middle), 5 μm (bottom). (TIF) [file pone.0336164.s005.tif]
